# Calibrating Hepatitis E Virus Serological Assays Using Asymptomatic Specimens Obtained in Japan

**DOI:** 10.1128/spectrum.02146-22

**Published:** 2022-09-20

**Authors:** Kazutaka Terahara, Tian-Cheng Li, Keiji Matsubayashi, Hidekatsu Sakata, Takanobu Kato, Atsushi Naganuma, Koji Ogawa, Koichi Honda, Jun Itakura, Noriyuki Akutsu, Hiroshi Tobita, Masaaki Korenaga, Tatsuya Kanto, Ryuichi Sugiyama, Ryosuke Suzuki, Isao Hamaguchi, Masanori Isogawa, Yoshimasa Takahashi

**Affiliations:** a Research Center for Drug and Vaccine Development, National Institute of Infectious Diseases, Tokyo, Japan; b Department of Virology II, National Institute of Infectious Diseases, Tokyo, Japan; c Central Blood Institute, Blood Service Headquarters, Japanese Red Cross Society, Tokyo, Japan; d Japanese Red Cross Hokkaido Block Blood Center, Sapporo, Japan; e Department of Gastroenterology, National Hospital Organization Takasaki General Medical Center, Gunma, Japan; f Department of Gastroenterology and Hepatology, Graduate School of Medicine, Hokkaido University, Hokkaido, Japan; g Department of Gastroenterology, Faculty of Medicine, Oita University, Oita, Japan; h Department of Gastroenterology and Hepatology, Musashino Red Cross Hospital, Tokyo, Japan; i Department of Gastroenterology and Hepatology, Sapporo Medical University School of Medicine, Hokkaido, Japan; j Department of Hepatology, Shimane University Faculty of Medicine, Shimane, Japan; k Hepatitis Information Center, Research Center for Hepatitis and Immunology, National Center for Global Health and Medicine, Chiba, Japan; l Research Center for Biological Products in the Next Generation, National Institute of Infectious Diseases, Tokyo, Japan; University of Sussex

**Keywords:** ELISA, HEV, IgA, IgM, immunoserology

## Abstract

This study aimed to calibrate hepatitis E virus (HEV) serological assays. We optimized the previously developed in-house HEV antibody enzyme-linked immunosorbent assay (ELISA) by setting the cutoff with an in-house serological performance panel consisting of broad HEV antibody titers and subtracting nonspecific background values for anti-HEV IgM, IgA, and IgG. We also compared the assay’s performance with that of commercial serological assay kits (four kits for IgM, one for IgA, and two for IgG). Although all serological assays readily detected HEV antibodies at high titers in the symptomatic hepatitis E population, considerable variations between assays were observed in the asymptomatic population. The in-house ELISA showed a higher sensitivity for HEV IgM, IgA, and IgG than the commercial kits and detected the seroconversion of HEV IgM and IgG earlier when testing a commercially available HEV seroconversion panel. The low sensitivity of the commercial kits was due to the high setting of the original cutoff, which was demonstrated by receiver operating characteristic analysis. However, the corrected cutoff value reduced assay specificity. Background subtraction is essential to achieve high specificity because the in-house ELISA without background subtraction reduced its specificity. These results indicate that asymptomatic specimens and background subtraction contribute to the optimization of HEV serological assays.

**IMPORTANCE** Accurate diagnosis of hepatitis E virus (HEV) infection is essential for public health surveillance and for preventing HEV-contaminated blood transfusion. Anti-HEV IgM or IgA is used as a reliable marker of recent HEV infection. However, considerable variability in the sensitivity and specificity of HEV antibody detection is observed among several commercially available assay kits. In addition, none of the HEV antibody detection methods have been approved by the U.S. Food and Drug Administration (FDA). Here, we show that the in-house enzyme-linked immunosorbent assay (ELISA) could detect HEV IgM and IgA more sensitively than commercial kits in the asymptomatic population. We also suggest that the assay performance of commercial kits might be improved by optimizing the cutoff and reducing nonspecific background noise. A sensitive serological (IgM or IgA) assay in addition to HEV RNA testing will contribute to accurate diagnosis of acute HEV infection because HEV RNA-positive duration is relatively short.

## INTRODUCTION

Hepatitis E virus (HEV), classified in the genus *Orthohepevirus* within the family *Hepeviridae*, is a major cause of acute hepatitis worldwide (1, 2). There are an estimated 20 million HEV infections worldwide every year, leading to an estimated 3.3 million symptomatic cases of hepatitis E, according to WHO fact sheets (updated 27 July 2021) (https://www.who.int/news-room/fact-sheets/detail/hepatitis-e). HEV that infects humans involves four major genotypes of the species *Orthohepevirus* A (HEV-A), and HEV infection in humans has shown two distinct epidemiological patterns (1–3). In developing countries, HEV-A genotypes 1 and 2 are transmitted between humans via the fecal-oral route through contaminated water. In industrialized countries, HEV-A genotypes 3 and 4 are transmitted zoonotically from animal reservoirs, such as swine, via ingestion of contaminated meat. Furthermore, transfusion-transmitted HEV infections have been documented (4–7). HEV infection is mostly asymptomatic but sometimes causes acute self-limiting hepatitis lasting 4 to 6 weeks (1, 2, 8). Importantly, chronic HEV infection is developed in immunocompromised individuals (1–3, 8). Thus, HEV infection has been considered a growing global health concern in recent years (3).

Accurate detection of HEV infection is crucial not only for public health surveillance but also for preventing HEV-contaminated blood transfusion. HEV infection can be diagnosed by directly detecting viral RNA or antigens or indirectly using anti-HEV antibodies. However, the appearance of these markers differs depending on the disease stage (9–14). HEV RNA and antigens indicate current infections. The HEV RNA assay is considered the gold standard for diagnosis because it is more sensitive than HEV antigen assays (15, 16). However, HEV RNA subsides soon after the appearance of symptoms (17, 18). In addition, RNA detection is available only in specialized laboratories. In contrast, indirect serological assays are more feasible in terms of cost and simplicity. HEV IgM and IgA indicate recent, but not necessarily current, infections (13, 19), and seroconversion is associated with viral clearance in the blood (20). Accordingly, approximately 20% of acute hepatitis E patients are seropositive but negative for HEV RNA (21). Problematically, considerable variability in the performance of HEV antibody detection has been observed among assays using commercially available kits (22–25), and none has been approved by the U.S. Food and Drug Administration (FDA).

We previously developed an in-house enzyme-linked immunosorbent assay (ELISA) for HEV antibodies using empty virus-like particles (VLPs) derived from HEV open reading frame 2 (ORF2) as an antigen (26). In this study, to calibrate serological assays for HEV infection, we optimized this in-house ELISA by setting the cutoff based on the receiver operating characteristic (ROC) analysis for a serological performance panel. To this end, we included the HEV RNA-positive asymptomatic population in the panel because it shows a broad range of HEV antibody titers (27). We then compared the assay performance of the in-house ELISA and commercial kits. Finally, we applied the same ROC analysis to the commercial kits to examine whether their original cutoff values were appropriate.

## RESULTS

### Preparation of in-house HEV serological performance panel.

**FIG 1:**
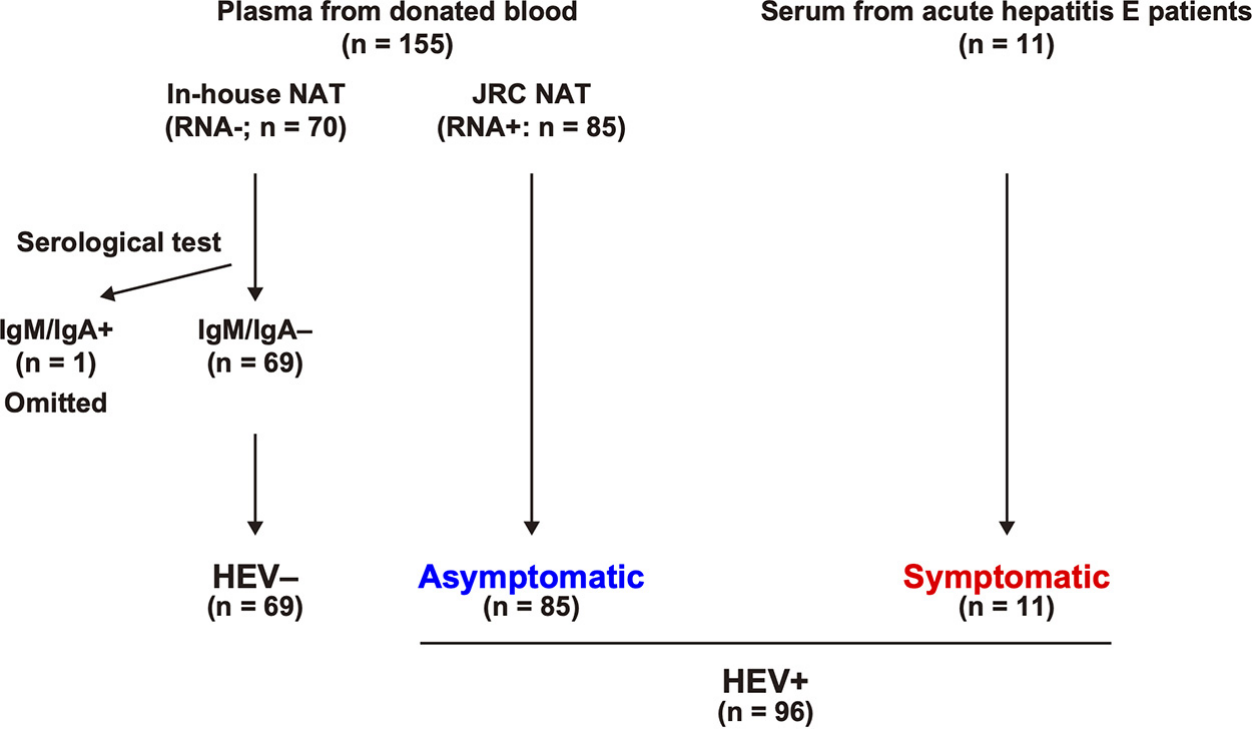
Flowchart for preparing the in-house performance panel. Plasma specimens from donated blood were collected from the Japanese Red Cross (JRC). Hepatitis E virus (HEV) RNA positivity (RNA+) and negativity (RNA−) were determined by the JRC nucleic acid amplification test (NAT) and the in-house NAT, respectively. Then, the HEV RNA-negative specimens were tested serologically using recomWell HEV IgG/IgM (Mikrogen), IgG/IgM anti-HEV EIA (Inst Immunol), and Immunis IgA anti-HEV EIA (Inst Immunol). Serum specimens from patients with acute hepatitis E were collected from six hospitals in Japan.

We prepared an in-house HEV serological performance panel consisting of plasma and serum samples (Fig. 1). For 70 plasma specimens that had not been tested by the Japanese Red Cross (JRC) nucleic acid amplification test (NAT), we performed an in-house NAT and confirmed that no HEV RNA was detected in any of these specimens. Consequently, all plasma specimens (*n* = 155) were divided into HEV RNA-positive (*n* = 85) and HEV RNA-negative (*n* = 70) groups. Seventy HEV RNA-negative specimens were serologically tested for HEV IgM and IgA using the Mikrogen and Inst Immunol kits, resulting in one HEV IgM/IgA double-positive (omitted from the analysis) and 69 HEV IgM/IgA double-negative specimens. Collectively, the in-house performance panel consisted of (i) the HEV-negative population (*n* = 69) that was negative for HEV RNA, HEV IgM, and HEV IgA, irrespective of HEV IgG; (ii) the asymptomatic population (*n* = 85) that was positive for HEV RNA; and (iii) the symptomatic population (*n* = 11) that was derived from acute hepatitis E patients.

### Assay performance of the in-house HEV antibody ELISA.

**FIG 2:**
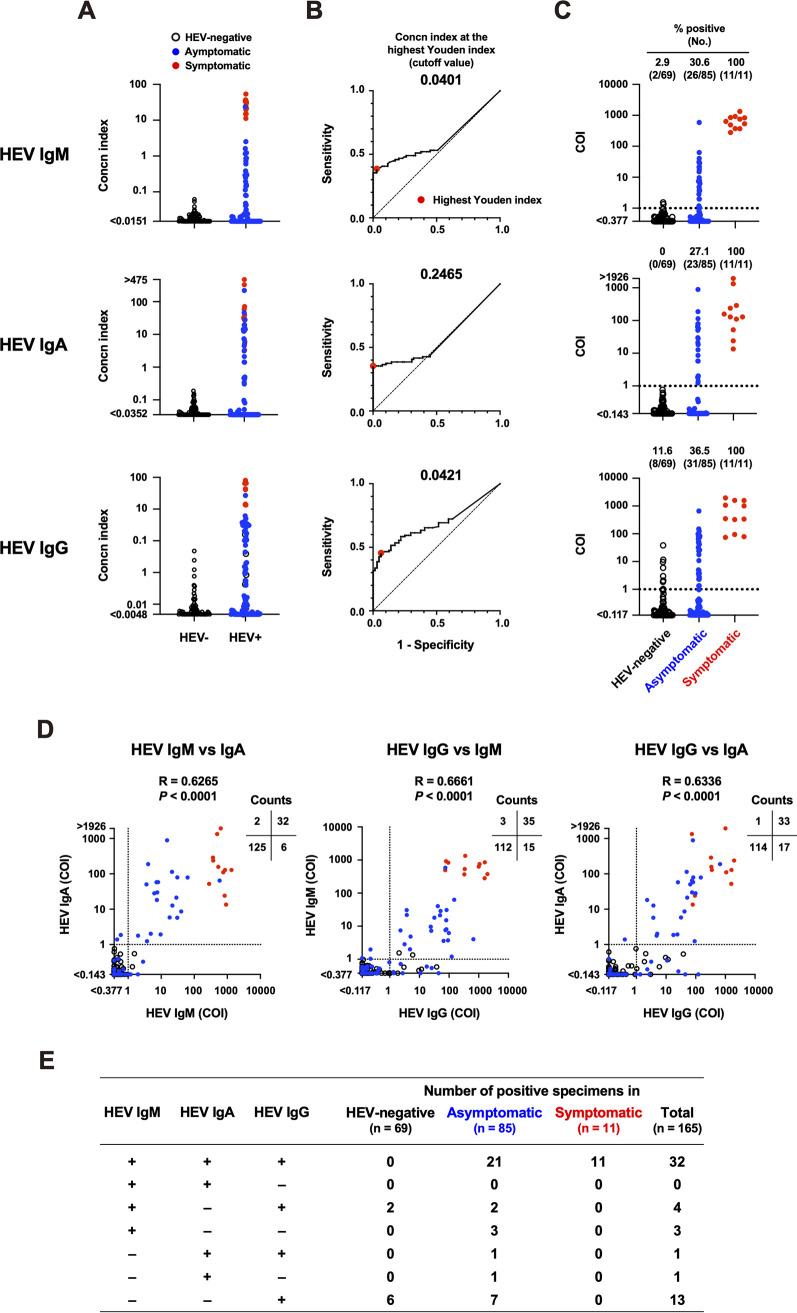
Assay performance of the in-house hepatitis E virus (HEV) antibody enzyme-linked immunosorbent assay (ELISA) using the in-house performance panel. (A) Concentration index of each specimen (HEV-negative, *n* = 69; asymptomatic, *n* = 85; symptomatic, *n* = 11) for HEV IgM, IgA, and IgG. For HEV IgM and IgA, HEV− and HEV+ groups consisted of 69 and 96 specimens, respectively. For HEV IgG, HEV− and HEV+ groups consisted of 64 and 101 specimens, respectively. (B) Receiver operating characteristic (ROC) analyses. The cutoff values at the highest Youden index (meaning optimal sensitivity and specificity, enlarged red dots) were 0.0401 for HEV IgM, 0.2465 for HEV IgA, and 0.0421 for HEV IgG. (C) HEV antibody titers based on cutoff index (COI) data among three populations. Dashed lines indicate the cutoff (COI = 1). (D) Comparison of the HEV antibody titers between antibody classes. Individual arithmetic data of the HEV-negative (black circles), asymptomatic (blue dots), and symptomatic (red dots) specimens are plotted. Dotted lines indicate the cutoff. The Spearman’s rank correlation coefficient was used for statical analysis. (E) The positive number of specimens in various combinations of HEV antibody classes.

**(i) Determination of the cutoff.** To determine the cutoff for the in-house ELISA, we performed ROC analyses using an in-house performance panel. For ROC analyses of HEV IgM and IgA, the concentration index data were divided into HEV-negative (*n* = 69) and HEV-positive (asymptomatic and symptomatic populations, *n* = 96) groups (Fig. 2A). However, because five specimens from the HEV-negative population were identified as HEV IgG-positive, suggestive of previous HEV infection, by the Mikrogen or Inst Immunol kits in our preliminary experiments (IDs N65-69; Table S1), they were included in the HEV-positive group when analyzing the cutoff for HEV IgG. The cutoff values of the concentration index were 0.0401, 0.2465, and 0.0421 for HEV IgM, HEV IgA, and HEV IgG, respectively (Fig. 2B). The concentration index of specimens was divided by the cutoff value to convert to the cutoff index (COI), meaning that a <1 COI was negative (Fig. 2C).

**(ii) Detection of HEV antibodies.** The in-house ELISA detected high titers of HEV IgM, IgA, and IgG in all symptomatic specimens (Fig. 2C; Table 1). The asymptomatic population was 30.6% HEV IgM-positive, 27.1% HEV IgA-positive, and 36.5% HEV IgG-positive with moderate titers. The HEV-negative population was negligible for HEV IgA, and only two specimens (IDs N7 and N66) were likely to be false-positive for HEV IgM (2.9%) near the cutoff (COI = 1.346 and 1.549, respectively) (Table S1), showing high specificities for HEV IgA and IgM. Eight HEV-negative specimens were HEV IgG-positive (11.6%) with relatively low titers; four of them were also HEV IgG-positive by the Mikrogen and/or Inst Immunol kits, and the other four specimens were HEV IgG-negative by the two commercial kits (Table S1). Since it is possible that the HEV-negative population contained previously infected subjects, it is difficult to determine whether these ambiguous specimens were true or false positive. We observed significant correlations between immunoglobulin classes (Fig. 2D). Detailed information is provided in Table S1. Notably, the two HEV IgM-positive specimens in the HEV-negative population (IDs N7 and N66) were also HEV IgG-positive (Fig. 2D and E; Table S1).

**TABLE 1:** Test performance evaluation of HEV serological assays using the in-house performance panel^*a*^

Target antibody	Assay method/kit		Applied cutoff	No. of positive specimens (%, 95% confidence interval)^*b*^		
				In HEV-negative (*n* = 69)	In asymptomatic (*n* = 85)	In symptomatic (*n* = 11)
HEV IgM	In-house	With background subtraction	ROC	2 (2.9, 0.5 to 10.0)	26 (30.6, 21.8 to 41.0)	11 (100, 74.1 to 100)
		Without background subtraction	ROC	9 (13.0, 7.0 to 23.0)	37 (43.5, 33.5 to 54.1)	11 (100, 74.1 to 100)
	Mikrogen		Original	0 (0, 0 to 5.3)	11 (12.9, 7.4 to 21.7)	11 (100, 74.1 to 100)
			ROC	13 (18.8, 11.4 to 29.6)	36 (42.4, 32.4 to 53.0)	11 (100, 74.1 to 100)
	Inst Immunol		Original	0 (0, 0 to 5.3)	5 (5.9, 2.5 to 13.0)	11 (100, 74.1 to 100)
			ROC	5 (7.2, 3.1 to 15.9)	32 (37.6, 28.1 to 48.3)	11 (100, 74.1 to 100)
	MP		Original	0 (0, 0 to 5.3)	5 (5.9, 2.5 to 13.0)	11 (100, 74.1 to 100)
			ROC	5 (7.2, 3.1 to 15.9)	23 (27.1, 18.8 to 37.3)	11 (100, 74.1 to 100)
	Wantai		Original	1 (1.4, 0.1 to 7.8)	10 (11.8, 6.5 to 20.3)	11 (100, 74.1 to 100)
			ROC	4 (5.8, 2.3 to 14.0)	21 (24.7, 16.8 to 34.8)	11 (100, 74.1 to 100)
HEV IgA	In-house	With background subtraction	ROC	0 (0, 0 to 5.3)	23 (27.1, 18.8 to 37.3)	11 (100, 74.1 to 100)
		Without background subtraction	ROC	29 (42.0, 31.1 to 53.8)	69 (81.2, 71.6 to 88.1)	11 (100, 74.1 to 100)
	Inst Immunol		Original	0 (0, 0 to 5.3)	12 (14.1, 8.3 to 23.1)	10 (90.9, 62.3 to 99.5)
			ROC	10 (14.5, 8.1 to 24.7)	55 (64.7, 54.1 to 74.0)	11 (100, 74.1 to 100)
HEV IgG	In-house	With background subtraction	ROC	8 (11.6, 6.0 to 21.2)	31 (36.5, 27.0 to 47.1)	11 (100, 74.1 to 100)
		Without background subtraction	ROC	17 (24.6, 16.0 to 36.0)	48 (56.5, 45.9 to 66.5)	11 (100, 74.1 to 100)
	Mikrogen		Original	3 (4.3, 1.2 to 12.0)	21 (24.7, 16.8 to 34.8)	11 (100, 74.1 to 100)
			ROC	7 (10.1, 5.0 to 19.5)	25 (29.4, 20.8 to 39.8)	11 (100, 74.1 to 100)
	Inst Immunol		Original	3 (4.3, 1.2 to 12.0)	23 (27.1, 18.8 to 37.3)	11 (100, 74.1 to 100)
			ROC	4 (5.8, 2.3 to 14.0)	26 (30.6, 21.8 to 41.0)	11 (100, 74.1 to 100)

a HEV, hepatitis E virus; ROC, receiver operating characteristic.

b The 95% confidence interval was determined by the hybrid Wilson/Brown method.

**(iii) Correlation between the HEV antibody titers and HEV RNA copy numbers.** Next, we compared HEV antibody titers with the HEV RNA copy numbers in the asymptomatic population and observed no significant correlations between the two variables (Fig. S1). These results are in accordance with those reported previously (16, 28).

### Assay performance of commercial serological assay kits.

**FIG 3:**
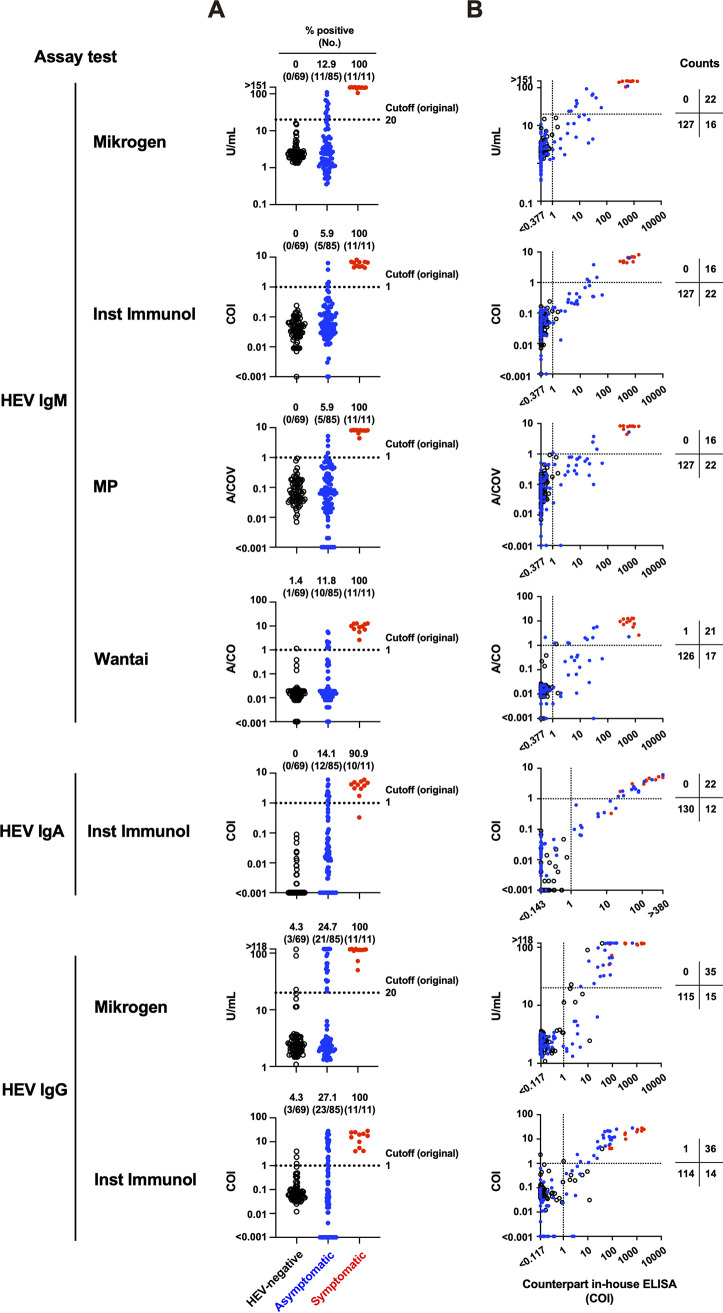
Assay performance of the commercial hepatitis E virus (HEV) antibody assay kits using the in-house performance panel. (A) HEV antibody titers among three populations. Dotted lines indicate the cutoff set by the manufacturer. (B) Comparison of the HEV antibody titers between the commercial kit and the counterpart in-house ELISA. Individual arithmetic data of the HEV-negative (black circles), asymptomatic (blue dots), and symptomatic (red dots) specimens are plotted. Dotted lines indicate the cutoff in each assay. A/CO, absorbance/cutoff.

Using the in-house performance panel, we tested the performance of the commercial HEV antibody assay kits (Fig. 3; Table 1). For HEV IgM, all four commercial kits (Mikrogen, Inst Immunol, MP, and Wantai) detected high antibody titers in all 11 symptomatic specimens. However, these commercial kits showed varied sensitivities when testing other populations; the Mikrogen and Wantai kits showed higher ratios of positive specimens (12.9 and 11.8%, respectively) than the Inst Immunol and MP kits (both 5.9%) in the asymptomatic population (Fig. 3A). In addition, whereas the Mikrogen, Inst Immunol, and MP kits detected no positive specimens in the HEV-negative population, only the Wantai kit detected one positive specimen (ID N7, absorbance/cutoff [A/CO] = 1.141) (Fig. 3A; Table S1), which was also positive in the in-house ELISA (COI = 1.346) (Fig. 3B; Table S1).

For HEV IgA, only one commercial kit (Inst Immunol) was used. The kit revealed 10 positive and one negative symptomatic specimens, although the titer of the latter was higher than that of any specimen in the HEV-negative population (Fig. 3A). Furthermore, 12 of 85 asymptomatic specimens were HEV IgA-positive. No HEV IgA-positive specimens were detected in the HEV-negative population.

For HEV IgG, two commercial kits (Mikrogen and Inst Immunol) were used. The two kits showed similar sensitivity (Fig. 3A), but positively detected specimens were, in part, different. There were two Mikrogen only positive specimens (IDs N66 and N68) and two Inst Immunol only positive specimens (IDs N65 and N69) in the HEV-negative population and two Inst Immunol only positive specimens (IDs AS45 and AS46) in the asymptomatic population (Table S1). These inconsistencies between the two kits were presumably due to the relatively low titers of HEV IgG (22.793 U/mL in the Mikrogen kit and 1.218 to 2.318 COI in the Inst Immunol kit), except for one specimen in the HEV-negative population (ID N68) showing a relatively high titer (89.618 U/mL) by the Mikrogen kit (Table S1).

Collectively, all HEV antibody-positive specimens detected by the commercial kits, except for one HEV IgM-positive specimen in the asymptomatic population using the Wantai kit (ID AS19) and one HEV IgG-positive specimen in the HEV-negative population using the Inst Immunol kit (ID N69) also detected as positive by the in-house ELISA (Table S1). However, a substantial number of specimens was positive by only the in-house ELISA (Fig. 3B; Table S1). These results indicate that every commercial kit exhibited lower sensitivity than the in-house ELISA.

### Sensitivity test using the HEV IgM quality control reagent.

**FIG 4:**
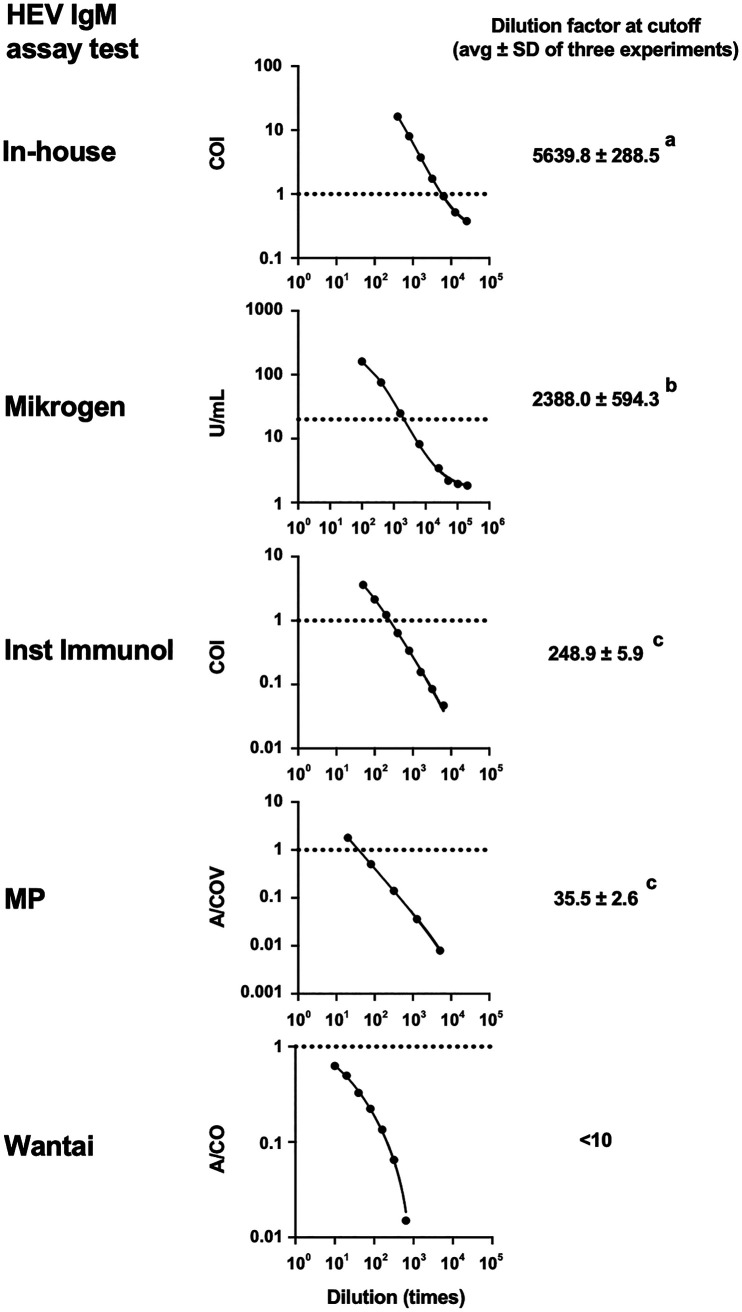
Evaluation of sensitivity for hepatitis E virus (HEV) IgM using the quality control reagent. Each panel is a representative regression curve of three tests. Dotted lines indicate the cutoff of assay tests. Dilution factors of the quality control reagent at the cutoff are denoted as the averages ± SD of three tests. Different letters denote statistical significance (*P < *0.001) determined by one-way analysis of variance (ANOVA) followed by Tukey’s multiple-comparison test where data from the Wantai IgM kit were not included. A/COV, absorbance/cutoff value.

Among HEV antibody classes, IgM and IgG, but not IgA, quality control reagents are available from the National Institute for Biological Standards and Control (NIBSC; Hertfordshire, UK). Here, we used the HEV IgM quality control reagent and compared the sensitivity among five assays (in-house, Mikrogen, Inst Immunol, MP, and Wantai) (Fig. 4). The in-house ELISA showed the highest sensitivity, detecting the quality control reagent up to 5,639.8 ± 288.5 (average ± standard deviation [SD] of three tests) times dilution, followed by the Mikrogen (2,388.0 ± 594.3 times dilution), Inst Immunol (248.9 ± 5.9 times dilution), MP (35.5 ± 2.6 times dilution), and Wantai (<10 times dilution). These results suggest that the in-house ELISA can detect HEV IgM more sensitively than the four commercial kits.

### Assay performance on the HEV seroconversion panel.

**FIG 5:**
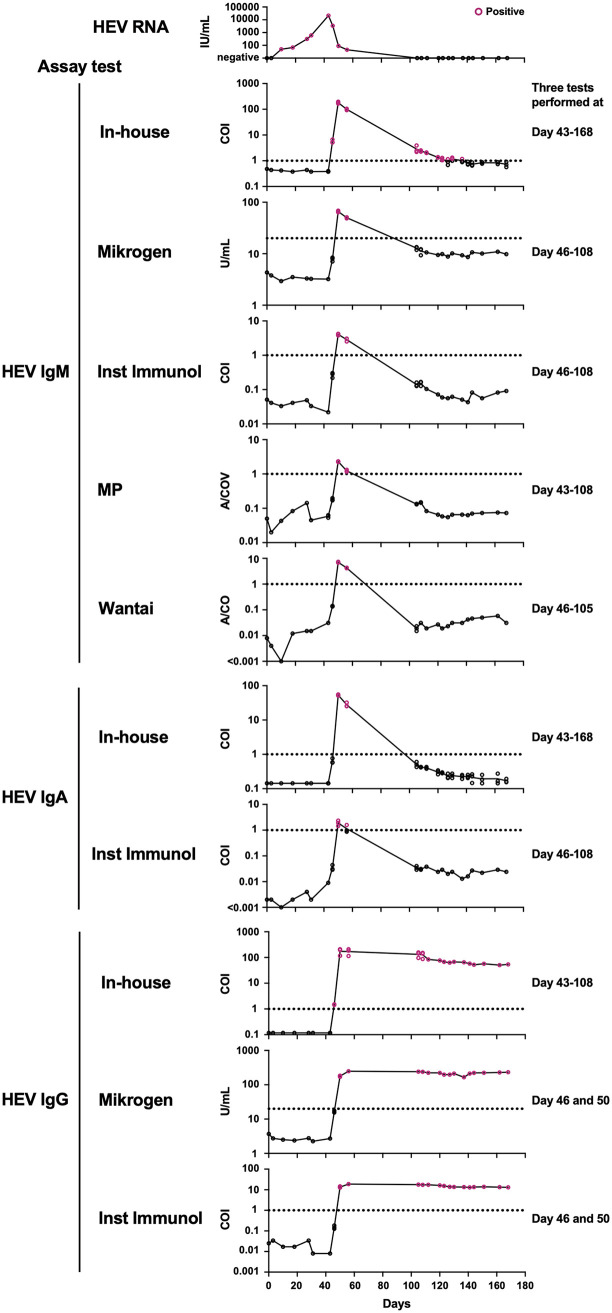
Changes in hepatitis E virus (HEV) antibody titers over time on the HEV seroconversion panel. Individual HEV antibody titers tested either once or three times are shown. Dotted lines indicate the cutoff. HEV RNA data are taken from the manufacturer (Biomex).

We also compared the assay performance of the various serological tests using a commercial HEV seroconversion panel, which allowed us to determine the timing of seroconversion relative to HEV RNA titers provided by the manufacturer (Fig. 5). Changes in HEV antibody titers over time were similar, but the timing and period of seropositivity were, in part, different between the in-house ELISA and commercial kits.

For HEV IgM, the in-house ELISA detected seroconversion on day 46 when HEV RNA started to decline, earlier than the Mikrogen, Inst Immunol, MP, and Wantai kits, which detected seroconversion on day 50. Furthermore, the in-house ELISA showed seropositivity until approximately day 130, whereas the four commercial kits exhibited seropositivity until day 56.

For HEV IgA, the in-house ELISA showed that only 2 days (days 50 and 56) were seropositive. The Inst Immunol kit showed similar results, but day 56 was ambiguous because only one of the three tests showed positive results.

For HEV IgG, the in-house ELISA detected seroconversion on day 46, earlier than the Mikrogen and Inst Immunol kits on day 50. Following seroconversion, all assays showed persistent seropositivity until the last sampling (day 168). Taken together, these results indicate that the in-house ELISA exhibited high assay performance for monitoring HEV seroconversion.

### Evaluation of the cutoff in the commercial assay kits.

**FIG 6:**
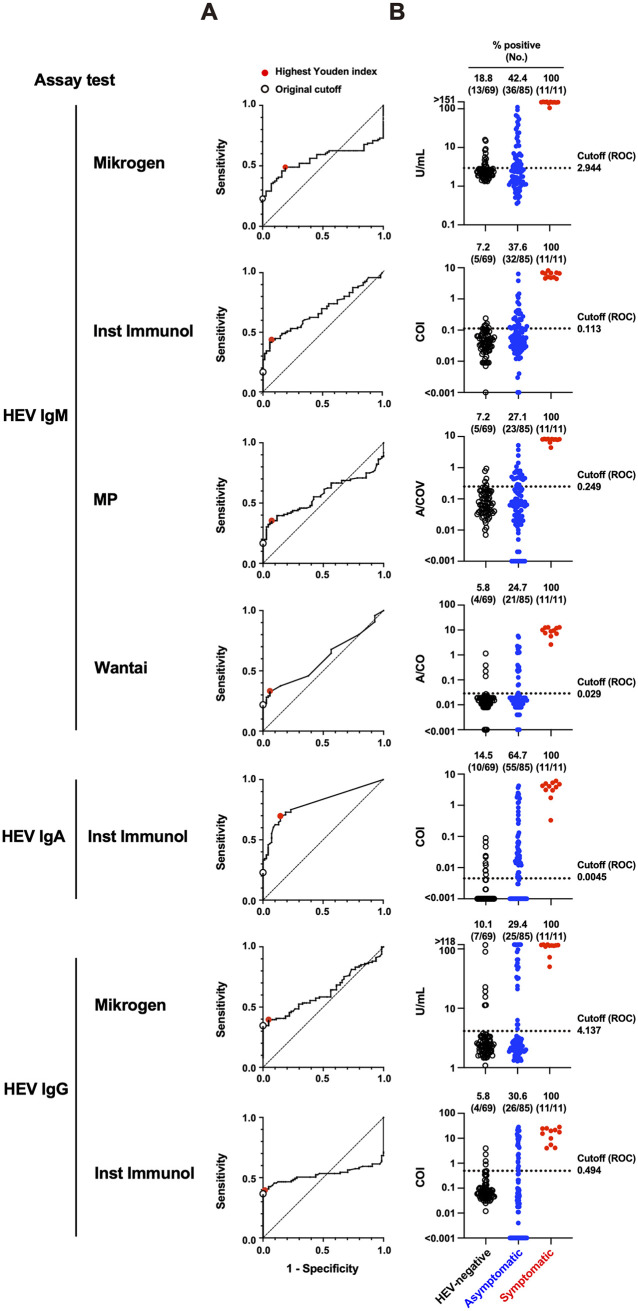
Evaluation of the cutoff setting of the commercial hepatitis E virus (HEV) antibody assay kits by receiver operating characteristic (ROC) analysis. (A) ROC analyses. Points indicating the highest Youden index (meaning optimal sensitivity and specificity) and the original cutoff are denoted as enlarged red dots and black circles, respectively. (B) HEV antibody titers among three populations. Dotted lines indicate the cutoff set by ROC analysis.

We speculated that the relatively low sensitivity of the commercial kits was due to the high original cutoff. Therefore, we applied ROC analyses to the commercial kits using data from the in-house performance panel. The ROC analyses demonstrated that all manufacturers’ original cutoff values could be lowered (Fig. 6A). When applying the cutoff values determined by the ROC analyses (ROC cutoff), the ratios of HEV IgM-positive and HEV IgA-positive were significantly increased, and those of HEV IgG-positive were slightly increased in the asymptomatic population (Fig. 6B; Table 1). However, the increased sensitivity was also concomitant with the increased ratios of false positives (positive in the HEV-negative population) compared with the in-house ELISA (Fig. 6B; Table 1).

### Background subtraction is essential for achieving high specificities in the in-house ELISA.

**FIG 7:**
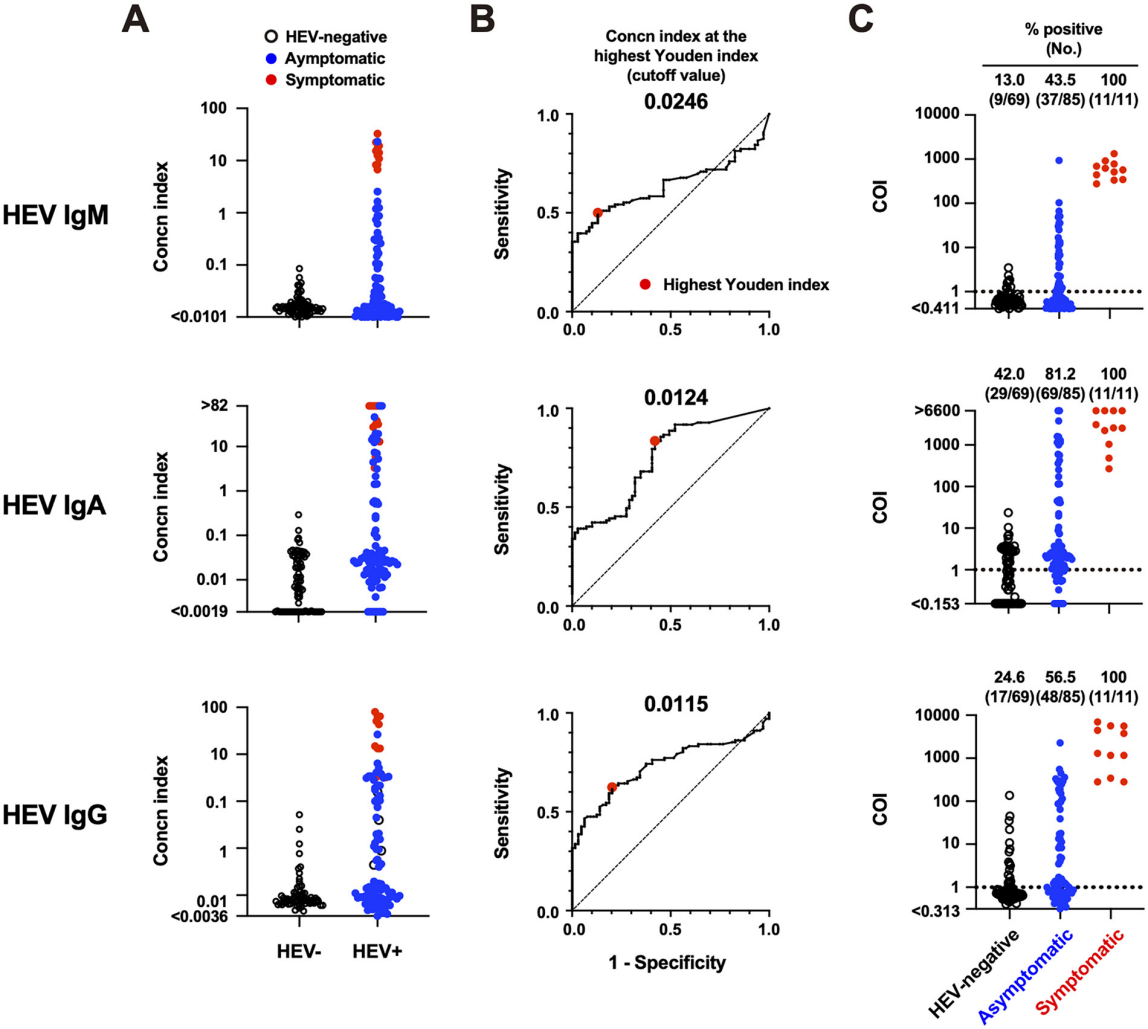
Evaluation of assay performance of the in-house hepatitis E virus (HEV) antibody enzyme-linked immunosorbent assay (ELISA) without background subtraction. The number of specimens analyzed were the same as in Fig. 2. (A) Concentration index of each specimen. (B) Receiver operating characteristic (ROC) analyses. The cutoff values at the highest Youden index (meaning optimal sensitivity and specificity, enlarged red dots) are 0.0246 for HEV IgM, 0.0124 for HEV IgA, and 0.0115 for HEV IgG. (C) HEV antibody titers based on cutoff index (COI) data among three populations. Dotted lines indicate the cutoff (COI = 1).

The in-house ELISA utilized a background subtraction protocol to repress the influence of nonspecific antibody reactions that could cause false positives. Here, we show the results when background subtraction was not performed (although only blank-well subtraction was performed) (Fig. 7). Similar to the procedure shown in Fig. 2, ROC analyses were performed on the concentration index data without background subtraction (Fig. 7A and B). Consequently, the ratios of HEV IgM-, IgA-, and IgG-positive specimens increased (Fig. 7C), indicating that significant nonspecific reactions occurred in any antibody class. Interestingly, one asymptomatic specimen detected as HEV IgM-positive using the Wantai kit (ID AS19; A/CO = 2.135) but not by the in-house ELISA (with background subtraction, COI = 0.540) (Fig. 3B; Table S1) became HEV IgM-positive (COI = 1.234) without background subtraction, indicating a false-positive result. Taken together, these results indicate that background subtraction is essential for achieving the high specificity of the in-house ELISA.

## DISCUSSION

One of the remarkable outcomes of the in-house ELISA was that it could detect HEV IgM, IgA, and IgG more sensitively than commercial kits in the asymptomatic population (Fig. 2 and 3; Table 1). In addition, it detected early seroconversion of HEV IgM and IgG and prolonged detection of HEV IgM compared with every commercial kit (Fig. 5). These results indicate that the in-house ELISA exhibited high assay performance.

To achieve optimal sensitivity and specificity, we calibrated the in-house ELISA as follows. First, we used an asymptomatic population with various HEV antibody titers, although the majority of available assays used in clinical practice were developed using blood specimens from symptomatic individuals (29). As we show here, HEV antibody responses between the symptomatic and HEV-negative populations were widely separated, not only in the in-house ELISA (Fig. 2) but also in the commercial kits (Fig. 3). Therefore, the cutoff might be imprecise if only two populations are evaluated. Second, we excluded background reactions by subtracting the absorbance of the noncoated control well from that of the antigen-coated well. This process is essential for reducing the rate of false positives (Fig. 7).

By optimizing the in-house ELISA, we detected three HEV IgM single-positive subjects and one HEV IgA single-positive subject, all of which were negative for HEV IgG, in the asymptomatic population (Fig. 2E), suggesting a very early phase of seroconversion. On the other hand, the in-house ELISA detected two HEV IgM-positive subjects in the HEV-negative population (Fig. 2). They were also HEV IgG-positive in the in-house ELISA; one (ID N7) was also HEV IgM-positive in the Wantai kit, and another (ID N66) was HEV IgG-positive in the Mikrogen kit, although all positive titers were near the cutoff (Table S1). Whereas weakly positive reactions of HEV IgM may mean very early or very late phase of seroconversion, as observed in Fig. 5, they might be cross-reactive false-positive because the in-house ELISA could not exclude the possibility of cross-reactive antibody reactions. Therefore, in clinical practice, such ambiguous specimens should be diagnosed not by a single serological parameter (only HEV IgM) but by a combination of other acute HEV infection-related parameters such as specific IgA or viral RNA, as described elsewhere (30).

The commercial kits tested in this study showed relatively low sensitivity in the asymptomatic population. ROC analyses revealed that the low sensitivity was due to the high setting of the original cutoff (Fig. 6A). Indeed, performance tests using the HEV IgM quality control reagent demonstrated that the original cutoff exhibited a significant margin from the detection limit (Fig. 4). However, simply applying the ROC cutoff reduced the specificity (Fig. 6B; Table 1) caused by nonspecific background noise. If nonspecific background noise can be significantly reduced, the cutoff can be optimized, resulting in excellent assay performance.

Despite the optimized assay performance of the in-house ELISA, some limitations remain. First, we did not selectively test specimens that were causative of nonspecific/cross-reactive antibody reactions, including rheumatoid arthritis and other pathogenic infections (31). It is expected that the cutoff can be optimized more practically by using such specimens for this purpose. However, it would still be difficult to distinguish between true positives and false positives because a substantial fraction of HEV infections is asymptomatic and may not be correctly diagnosed as HEV infection due to the relatively short HEV RNA-positive period. Second, we did not evaluate the influence of HEV genotype on assay performance. Although HEV has one serotype, differences in the genotype of antigens can affect detection sensitivity (32). Since HEV-A genotype 3 is predominantly detected in donated blood (asymptomatic) in Japan (27, 33, 34), we used HEV-A genotype 3-derived VLPs as antigens in the in-house ELISA. In contrast, the MP kit might not perform well in the asymptomatic population in Japan because it uses antigens from HEV-A genotype 1 (35) (Table 2). Third, there were a substantial number of HEV IgG-positive asymptomatic subjects that were HEV IgM- and IgA-negative, although they were HEV RNA-positive (Fig. 2). This might reflect competitive inhibition by IgG at antigen-binding sites, which often causes false negatives for IgM (36). To overcome this problem, Yu et al. introduced an IgM class capture system in which competing IgG and IgA in the specimens were eliminated at the beginning of the assay, enhancing the reaction between HEV IgM and HEV antigens (37). Several HEV IgM assay kits based on the IgM class capture principle are now commercially available; therefore, we used the Wantai kit. Surprisingly, the in-house ELISA (an antigen-solid indirect format but not IgM-capture or IgG-absorbent formats) showed higher ratios of HEV IgM-positive specimens than the Wantai kit in the asymptomatic population, even when the ROC cutoff was applied to the Wantai kit (Table 1). Further comparisons with other HEV IgM capture assays are required. Fourth, we did not examine whether the sample status, either serum or plasma, could affect the results. Because all serum and plasma specimens were equally analyzed by all serological assays performed in this study, our main massage is not affected by this shortcoming. However, the comparison between serum and plasma may be required when the in-house ELISA is approved for diagnosis.

**TABLE 2:** Information for HEV antibody assays tested in this study^*a*^

Manufacturer	Product	Intended use	Antigen (origin)	Test vol (dilution)	Cutoff (unit)
In-house		Research	ORF2 (genotype 3)	50 μL (1:100)	Negative: <1 (COI)
					Positive: ≥1 (COI)
Mikrogen	RecomWell HEV IgG/IgM	Research	ORF2 (genotype 1 and 3)	100 μL (1:100)	Negative: <20 (U/mL)
					Borderline: ≤20 to ≤24 (U/mL)
					Positive: >24 (U/mL)
Inst Immunol	IgG/IgM anti-HEV EIA	Research	ORF2 (genotype 4)	50 μL (1:100)	Negative: <1 (COI)
	Immunis IgA anti-HEV EIA	Diagnosis^*b*^			Positive: ≥1 (COI)
MP	MP Diagnostics HEV IgM ELISA 3.0	Research	ORF2 (genotype 1)	210 μL (1:20)	Negative: <0.4 + NRC (COV)
					Positive: ≥0.4 + NRC (COV)
Wantai	Wantai HEV-IgM ELISA	Research	ORF2 (highly conserved region)	110 μL (1:10)	Negative: <1 (A/CO)
					Positive: ≥1 (A/CO)

a A/CO, absorbance/cutoff; A/COI, absorbance/cutoff index; COI, cutoff index; COV, cutoff value; EIA, enzyme immunoassay; ELISA, enzyme-linked immunosorbent assay; HEV, hepatitis E virus; NRC, nonreactive control mean; ORF, open reading frame.

b Approved in Japan.

In summary, asymptomatic specimens and background subtraction contributed to optimizing HEV serological assays to ensure that the in-house ELISA demonstrated higher assay performance than the commercial kits. However, a combination of serological (IgM or IgA) and RNA testing, as well as testing paired sera, are still needed for the accurate diagnosis of HEV infection because approximately 70% of asymptomatic subjects were negative for HEV IgM or IgA.

## MATERIALS AND METHODS

### Blood collection.

Plasma specimens (*n* = 155) from donated blood that had been determined to be ineligible for transfusion were provided by the JRC Blood Centers (Tokyo, Japan). These specimens were collected from 2006 to 2020 in Japan. The plasma specimens included 85 HEV RNA-positive specimens that had been determined by the HEV NAT using a previously described method (33), consisting of 69 HEV-A genotype 3 and 16 HEV-A genotype 4 specimens. The other 70 plasma specimens were not tested by the NAT. They were ineligible for transfusion on the basis of various criteria (38), but were, at least, all negative for HBsAg, anti-HBc antibody, HBV DNA, anti-hepatitis C virus (HCV) antibody, HCV RNA, anti-HIV-1/2 antibody, HIV-1/2 RNA, anti-Treponema pallidum antibody, anti-human T-cell leukemia virus type-1/2 antibody, and human parvovirus B19 Ag. Serum specimens from patients with acute hepatitis E patients (*n* = 11) were provided from six hospitals in Japan (Hokkaido University Hospital, Sapporo Medical University Hospital, Takasaki General Medical Center, Japanese Red Cross Musashino Hospital, Shimane University Hospital, and Oita University Hospital) from 2018 to 2020. All studies were approved by the Institutional Review Board of the National Institute of Infectious Diseases (approval nos. 1082, 1083, and 1359) and were performed in accordance with the Declaration of Helsinki. All volunteers provided written informed consent prior to enrollment. All specimens were aliquoted and stored at −80°C until use.

### Commercially available specimens for HEV serological assays.

A quality control reagent for anti-HEV IgM (QCRHEVIgMQC1, code 17/B728) was obtained from the NIBSC. An HEV seroconversion panel, consisting of plasma of 23 time-points (to day 168) from one donor infected with HEV-A genotype 3, was obtained from Biomex (catalog no. SCP-HEV-006a, Heidelberg, Germany). All specimens were aliquoted and stored at −80°C until use.

### In-house HEV NAT.

Total RNA was extracted from 140 μL of plasma using the QIAamp viral RNA minikit (Qiagen, Hilden, Germany). Purified RNA was eluted in a 40-μL volume and was subjected to in-house quantitative and qualitative reverse transcription (RT)-PCR. The TaqMan Fast virus 1-step Master Mix (Thermo Fisher Scientific, Waltham, MA) was used for 20 μL of the RNA template in a total 40-μL reaction volume. The forward primer (JVHEVF; 5′-GGTGGTTTCTGGGGTGAC-3′), reverse primer (JVHEVR; 5′-AGGGGTTGGTTGGATGAA-3′), and probe (JVHEVP; 6-carboxyfluorescein [FAM]–5′-TGATTCTCAGCCCTTCGC-3′–6-carboxytetramethylrhodamine [TAMRA]) were designed as described previously (39). PCR conditions included reverse transcription (RT) (50°C for 5 min), followed by RT inactivation/initial denaturation (95°C for 20 s) and 45 cycles of denaturation (95°C for 3 s) and annealing/extension (60°C for 30 s) on the AriaMx real-time PCR system (Agilent, Santa Clara, CA). RNA extracted from HEV83.2 (accession no. AB740232) was used as the reference. The detection limit was 14.3 RNA copies/mL plasma.

### Commercial HEV antibody assay kits.

Five commercial HEV antibody assay kits were used as follows: (i) recomWell HEV IgG/IgM (Mikrogen Diagnostik, Neuried, Germany); (ii) IgG/IgM anti-HEV EIA (Institute of Immunology [Inst Immunol], Tokyo, Japan); (iii) MP Diagnostics HEV IgM ELISA 3.0 (MP Biomedicals Asia Pacific, Singapore); (iv) Wantai HEV-IgM ELISA (Wantai Biological Pharmacy, Beijing, China); and (v) Immunis IgA anti-HEV EIA (Inst Immunol). Only Immunis IgA anti-HEV EIA has been approved as a clinical diagnostic reagent for HEV infection in Japan, and the others are intended for research use. All commercial kits, except for the Wantai kit, are in an antigen-solid format, whereas the Wantai kit is in an anti-IgM antibody-solid format. Fundamental information for these commercial kits is presented in Table 2. These commercial kits were used according to the manufacturer’s instructions, except for the sample volume when testing the anti-HEV IgM quality control. In testing the anti-HEV IgM quality control, a 50-μL sample volume was used for every assay to equalize the amount of anti-HEV IgM in a given dilution, although the use of a 50-μL sample volume was irregular in the Mikrogen, MP, and Wantai kits. Furthermore, although the Mikrogen kits officially set the cutoff of the sample arithmetic values as follows: <20 U/mL negative, 20 to 24 U/mL borderline, and >24 U/mL positive; we regarded ≥20 U/mL positive in this study. In addition, although the MP kit, which is for qualitative tests, officially sets the cutoff value (COV) as 0.4 plus the absorbance of the nonreactive control, we calculated the arithmetic value (the absorbance of a specimen to the COV, A/COV) to allow quantitative evaluation. Unless otherwise noted, serological assays using commercial kits were initially performed in a single well for each sample. If the initial arithmetic value of samples were near the tentative cutoff (ranging from 50 to 200% of the cutoff), two more tests were repeated (a total of three replicates), and the average arithmetic value of two or three matched positive/negative decisions was used.

### In-house HEV antibody ELISA.

A previously developed in-house HEV antibody ELISA (26) was modified in this study. VLPs consisting of recombinant ORF2 from HEV-A genotype 3 were generated and purified as previously described (26). VLPs were prepared at a concentration of 2 μg/mL with carbonate buffer (15 mM Na_2_CO_3_, 35 mM NaHCO_3_, pH 9.6) and half of the test wells of a 96-well polystyrene flat plate (F96 Maxisorp Nunc-Immuno plate, Thermo Fisher Scientific) were coated with 50 μL VLPs solution overnight at 4°C. After washing with phosphate-buffered saline containing 0.05% Tween 20 (PBST), all VLP-coated and noncoated control wells were blocked with ChonBlock blocking/sample dilution buffer (Chondrex Inc., Redmond, WA) for 1 h at room temperature. After washing with PBST, 50 μL of diluted (1:100) plasma or serum samples with ChonBlock blocking/sample dilution buffer was added to both VLP-coated and noncoated control wells and incubated for 2 h at room temperature. After washing with PBST, 100 μL of diluted (1:5,000) horseradish peroxidase (HRP)-conjugated secondary antibodies (goat anti-human IgM, IgA, or IgG; all from SouthernBiotech, Birmingham, AL) with ChonBlock detection antibody dilution buffer (Chondrex) was added to all wells and incubated for 1 h at room temperature. After washing with PBST, HRP activity was visualized by the addition of one-step Ultra TMB-ELISA (Thermo Fisher Scientific) and stopped by the addition of 2 N sulfuric acid. Absorbance values were measured at 450/630 nm using an iMark microplate reader (Bio-Rad Laboratories, Hercules, CA). The net absorbance value was calculated by subtracting the absorbance value of the noncoated control well from that of the corresponding VLP-coated well. The serum from a symptomatic patient (ID S1; Table S1), in which all HEV IgM, IgA, and IgG were detected by commercial serological kits (Mikrogen HEV IgG/IgM, Inst Imunol HEV IgG/IgM, and Inst Immunol HEV IgA) in the preliminary experiment, was used as a reference, and serial dilutions were added to every plate. A calibration curve between net absorbance and the concentration index (6,400 [an arbitrary number]/dilution factor) was created for each plate using a nonlinear regression model in Prism 9 software (GraphPad Software, Inc., San Diego, CA). The concentration index of each sample was obtained from the corresponding calibration curve. We tentatively performed an ROC analysis based on a single well from each sample using Prism 9 software. When the concentration index ranged 50 to 200% of the tentative cutoff value, the corresponding specimens were further tested twice (resulting in a total of three tests), and the average concentration index of the three tests was used. We again performed an ROC analysis based on the corrected data.

### Data analyses.

ROC analysis, interpolation from a calibration curve obtained by nonlinear regression model, the hybrid Wilson/Brown method, Spearman’s rank correlation coefficient, and one-way analysis of variance (ANOVA) followed by Tukey’s multiple-comparison test were performed using Prism 9 software. A *P* value of <0.05 was considered statistically significant.
