# Supplementary material for: Calibrating Hepatitis E Virus Serological Assays Using Asymptomatic Specimens Obtained in Japan
Source: Microbiol Spectr. 2022 Sep 20;10(5):e02146-22. doi: 10.1128/spectrum.02146-22 (PMC9603090; doi:10.1128/spectrum.02146-22)
Supplement: Supplemental file 1 — Table S1 and Fig. S1. Download spectrum.02146-22-s0001.pdf, PDF file, 0.1 MB [file spectrum.02146-22-s0001.pdf]

Supplementary TABLE 1 HEV antibody titers in all serological assays tested

Population	Specimen ID	HEV IgM					HEV IgA		HEV IgG		
		In-house (COI)	Mikrogen (U/mL)	Inst Immunol (COI)	MP (A/COV)	Wantai (A/CO)	In-house (COI)	Inst Immunol (COI)	In-house (COI)	Mikrogen (U/mL)	Inst Immunol (COI)
HEV-negative (n = 69)	N1	<0.377	3.701	0.064	0.062	0.015	0.249	0.008	0.689***	3.772	0.037
	N2	0.386	8.517	0.085	0.072	0.019	0.763	0.012	0.970***	3.364	0.171
	N3	<0.377	3.344	0.059	0.072	0.008	<0.143	0.067	0.897***	3.398	0.025
	N4	<0.377	2.586	0.164	0.047	0.008	<0.143	0.008	0.121	3.297	0.136
	N5	0.433	2.408	0.049	0.100	0.008	0.235	0.000	<0.117	2.549	0.087
	N6	<0.377	2.363	0.111	0.137	0.011	0.341	0.000	<0.117	2.889	0.045
	N7	1.346***	15.890***	0.064	0.783***	1.141***	0.235	0.002	5.921	15.612***	0.331
	N8	<0.377	2.453	0.035	0.022	0.015	<0.143	0.000	0.121	3.602	0.059
	N9	<0.377	2.230	0.083	0.065	0.015	<0.143	0.000	0.130	3.331	0.052
	N10	0.718	2.363	0.049	0.050	0.019	0.209	0.014	<0.117	2.957	0.080
	N11	0.470	4.771	0.138	0.187	0.000	0.235	0.000	<0.117	3.161	0.080
	N12	<0.377	1.873	0.025	0.037	0.011	0.182	0.002	0.139	3.466	0.059
	N13	0.457	3.701	0.104	0.187	0.015	<0.143	0.000	0.139	2.345	0.101
	N14	<0.377	2.453	0.056	0.184	0.027	<0.143	0.000	<0.117	2.447	0.052
	N15	<0.377	2.542	0.030	0.037	0.019	0.182	0.000	<0.117	2.821	0.345
	N16	0.545	1.650	0.020	0.050	0.019	<0.143	0.000	0.149	3.331	0.094
	N17	<0.377	2.230	0.069	0.226	0.000	0.196	0.000	0.200	2.345	0.038
	N18	0.500***	4.103	0.020	0.040	0.008	0.380	0.022	11.709	2.447	0.031
	N19	0.386	1.962	0.077	0.162	0.015	0.209	0.000	0.191	2.821	0.012
	N20	0.598***	3.300	0.041	0.194	0.011	0.328	0.002	0.167	2.311	0.888***
	N21	<0.377	1.516	0.054	0.017	0.015	0.169	0.000	0.167	2.481	0.052
	N22	<0.377	2.007	0.009	0.012	0.019	<0.143	0.000	0.144	1.971	0.031
	N23	<0.377	2.809	0.030	0.035	0.015	0.156	0.000	<0.117	2.209	0.038
	N24	<0.377	2.586	0.009	0.080	0.023	0.182	0.000	0.130	3.195	0.436
	N25	<0.377	1.561	0.025	0.037	0.000	0.249	0.000	0.532***	3.670	0.031
	N26	<0.377	2.765	0.046	0.179	0.011	0.619***	0.047	<0.117	2.855	0.101
	N27	<0.377	1.338	0.009	0.100	0.008	0.367	0.006	<0.117	2.515	0.059
	N28	<0.377	1.873	0.046	0.102	0.011	<0.143	0.000	0.167	2.719	0.059
	N29	<0.377	1.472	0.012	0.025	0.011	<0.143	0.000	<0.117	2.345	0.059
	N30	<0.377	1.962	0.030	0.025	0.015	<0.143	0.000	<0.117	2.515	0.129
	N31	<0.377	1.828	0.033	0.040	0.019	<0.143	0.000	<0.117	2.583	0.038
	N32	<0.377	1.695	0.038	0.042	0.023	<0.143	0.000	0.257	2.345	0.488***
	N33	<0.377	1.561	0.000	0.035	0.011	0.328	0.000	1.860	19.321**	0.209
	N34	<0.377	2.720	0.017	0.062	0.011	0.169	0.000	<0.117	2.719	0.045
	N35	<0.377	1.427	0.007	0.007	0.019	0.209	0.000	<0.117	1.801	0.073
	N36	<0.377	2.096	0.014	0.010	0.015	<0.143	0.000	<0.117	2.005	0.052
	N37	0.589	1.873	0.090	0.035	0.015	<0.143	0.004	<0.117	2.243	0.080
	N38	0.633	5.128	0.035	0.358	0.019	0.235	0.000	0.199	2.481	0.059
	N39	0.473	1.338	0.009	0.027	0.008	<0.143	0.000	0.139	1.971	0.080
	N40	0.501	2.051	0.077	0.072	0.011	0.178	0.000	<0.117	2.481	0.073
	N41	0.781***	2.453	0.240	0.936**	0.015	<0.143	0.089	<0.117	1.767	0.059
	N42	0.501	2.453	0.041	0.032	0.015	<0.143	0.000	0.220	1.869	0.080
	N43	<0.377	2.363	0.022	0.020	0.011	<0.143	0.000	<0.117	2.311	0.045
	N44	0.633	1.695	0.104	0.032	0.019	<0.143	0.004	0.155	1.699	0.059
	N45	<0.377	1.918	0.054	0.045	0.011	<0.143	0.000	0.145	1.903	0.080
	N46	0.404	1.382	0.009	0.040	0.224	<0.143	0.000	<0.117	2.209	0.080
	N47	<0.377	2.082	0.028	0.060	0.011	<0.143	0.000	<0.117	1.733	0.066
	N48	0.523***	2.776	0.028	0.065	0.011	<0.143	0.000	<0.117	1.665	0.058
	N49	0.589	2.397	0.049	0.109	0.019	<0.143	0.000	<0.117	1.869	0.104
	N50	0.530	2.019	0.049	0.045	0.019	<0.143	0.000	0.155	1.495	0.073
	N51	0.971***	5.678	0.117	0.179	0.019	<0.143	0.000	0.129	1.597	0.043
	N52	0.501	2.524	0.062	0.284	0.019	<0.143	0.000	0.172	2.107	0.085
	N53	0.530	1.893	0.049	0.234	0.023	<0.143	0.000	0.150	2.107	0.101
	N54	<0.377	2.271	0.030	0.107	0.000	<0.143	0.000	<0.117	2.073	0.039
	N55	0.501	14.856***	0.093	0.445	0.011	<0.143	0.000	0.129	1.835	0.139
	N56	<0.377	2.334	0.062	0.152	0.011	<0.143	0.000	0.215	1.971	0.039
	N57	0.417	2.650	0.035	0.027	0.019	<0.143	0.000	0.330	1.495	0.058
	N58	0.516	2.208	0.022	0.037	0.141	<0.143	0.000	0.358	1.733	0.062
	N59	0.487	2.587	0.104	0.149	0.023	<0.143	0.000	0.446***	1.631	0.058
	N60	0.574	2.713	0.020	0.132	0.023	<0.143	0.000	2.996	11.356***	0.197
	N61	0.459	2.208	0.054	0.060	0.019	<0.143	0.024	0.228***	1.597	0.035
	N62	0.390	2.082	0.028	0.072	0.000	0.178	0.000	0.353***	1.631	0.043
	N63	0.648	2.839	0.146	0.119	0.008	0.155	0.000	0.172	1.088	0.046
	N64	<0.377	2.082	0.022	0.040	0.015	<0.143	0.000	0.129	1.461	0.031
	N65	<0.377	2.809	0.033	0.097	0.008	0.209	0.000	1.029***	9.906***	1.218***
	N66	1.549***	9.142	0.109	0.233	0.011	0.552	0.000	2.058	22.793**	0.331
	N67	0.584	7.090	0.064	0.154	0.384	0.539	0.000	38.172	116.805	3.950
	N68	0.457	2.720	0.041	0.172	0.008	0.430***	0.004	9.565	89.618	0.464
	N69	<0.377	2.019	0.009	0.052	0.011	<0.143	0.000	<0.117	1.461	2.278**
	AS1	<0.377	2.354	0.132	0.294	0.008	<0.143	0.000	2.722	5.300***	0.000
	AS2	<0.377	0.646	0.030	0.062	0.023	<0.143	0.004	0.398***	2.651	0.000
	AS3	0.717	1.754	0.093	0.453	0.015	0.199	0.000	2.323	1.325	0.000
	AS4	<0.377	1.308	0.108	0.070	0.015	0.187	0.034	0.131	2.005	0.000
	AS5	1.031***	2.323	0.147	1.085**	0.004	<0.143	0.000	0.224	1.461	0.000
	AS6	<0.377	0.846	0.021	0.124	0.004	<0.143	0.000	0.126	1.325	0.000
	AS7	0.805***	1.262	0.048	0.075	0.015	<0.143	0.006	0.970***	1.971	0.024
	AS8	<0.377	0.677	0.060	0.037	0.011	<0.143	0.014	<0.117	1.495	0.000
	AS9	<0.377	0.508	0.042	0.080	0.015	<0.143	0.020	<0.117	2.159	0.000
	AS10	<0.377	0.492	0.024	0.182	0.015	<0.143	0.039	0.153	3.111	0.000
	AS11	<0.377	0.538	0.042	0.095	0.019	<0.143	0.012	<0.117	2.781	0.000
	AS12	<0.377	1.092	0.051	0.022	0.008	<0.143	0.014	<0.117	3.038	0.000
	AS13	2.831***	1.692	0.114	0.204	0.217	0.327	0.046	3.143***	5.306	0.048
	AS14	<0.377	0.354	0.012	0.020	0.011	<0.143	0.037	<0.117	3.294	0.000
	AS15	<0.377	1.138	0.054	0.075	0.011	<0.143	0.020	<0.117	2.855	0.000
	AS16	0.463	1.523	0.072	0.088	0.023	1.388***	0.622***	0.404	3.403	0.000
	AS17	<0.377	1.123	0.057	0.065	0.019	<0.143	0.007	0.153	2.452	0.000
	AS18	<0.377	0.985	0.027	0.000	0.019	<0.143	0.000	<0.117	2.452	0.000
	AS19	0.540	6.063	0.045	0.459***	2.135	0.174	0.033	26.022	97.786	4.236
	AS20	<0.377	1.407	0.084	0.058	0.004	<0.143	0.056	44.470	32.315	11.107
	AS21	1.196***	3.686	0.151	0.453	1.237**	<0.143	0.069	122.601	122.708	12.055
	AS22	15.074	19.137**	0.682***	0.580***	2.034***	887.235	6.077	80.095	52.077	6.159
	AS23	6.208	11.322***	0.198	0.283	0.240	28.215	1.785***	98.489	66.130	11.409
	AS24	3.713	11.148***	0.274	0.415	0.061	1.237***	0.099	92.035	118.207	9.300
	AS25	4.042	23.755**	0.433	0.785***	1.224***	187.677	4.219	661.528	> 128	28.398
	AS26	7.608	45.659	0.253	0.677**	0.335	29.870	1.259***	75.314	65.691	21.225
	AS27	<0.377	10.802***	0.057	0.508***	0.008	0.353	0.024	10.849	57.713	1.084***
	AS28	<0.377	1.215	0.018	0.000	0.011	<0.143	0.005	0.387	2.855	0.000
	AS29	<0.377	0.615	0.027	0.063	0.008	<0.143	0.005	<0.117	2.086	0.000

	AS30	<0.377	0.831	0.012	0.083	0.011	<0.143	0.029	<0.117	2.745	0.000
	AS31	0.620	2.477	0.042	0.080	0.015	<0.143	0.016	0.175	2.781	0.000
	AS32	<0.377	6.908	0.036	0.028	0.011	<0.143	0.007	<0.117	2.159	0.000
	AS33	<0.377	0.846	0.027	0.008	0.008	<0.143	0.000	<0.117	2.306	0.000
	AS34	<0.377	1.523	0.039	0.043	0.015	<0.143	0.003	<0.117	2.123	0.000
	AS35	0.409	3.108	0.102	0.015	0.015	<0.143	0.001	1.334***	1.793	0.381
	AS36	0.523	3.508	0.123	0.160	0.015	<0.143	0.026	0.447***	2.745	0.000
	AS37	<0.377	2.231	0.036	0.063	0.015	<0.143	0.000	<0.117	2.269	0.000
	AS38	<0.377	1.046	0.003	0.002	0.019	<0.143	0.020	<0.117	2.379	0.000
	AS39	21.283	3.460	1.102**	0.200	0.030	114.084	3.673	49.495	89.149	20.182
	AS40	3.579	6.209	0.226	0.263	1.275**	50.288	1.999***	65.105	122.964	21.845
	AS41	28.790	63.266	0.348	2.458	2.079***	21.284	1.195**	51.688	116.560	5.179
Asymptomatic (n = 85)	AS42	7.227	32.078	0.230	0.430	0.065	57.788	2.499***	25.025	45.416	7.843
	AS43	8.031	38.529	0.195	0.694***	0.393	57.997	2.281	81.308	>128	21.003
	AS44	62.834	29.735***	0.389	0.498	0.282	78.684	1.656***	148.960	>128	25.395
	AS45	9.636	17.233**	0.331	0.792**	0.126	1.936***	0.063	24.068	6.295	1.618***
	AS46	21.866	14.297***	0.790***	0.478	0.240	12.648	0.841***	3.745	4.501	2.318
	AS47	4.863	24.102***	0.213	0.798**	0.332	2.024***	0.113	5.032	21.150**	1.187***
	AS48	<0.377	0.384	0.029	0.028	0.008	<0.143	0.000	<0.117	2.489	0.626***
	AS49	<0.377	0.767	0.082	0.043	0.008	<0.143	0.000	<0.117	1.647	0.086
	AS50	<0.377	2.446	0.059	0.058	0.011	<0.143	0.000	<0.117	2.306	0.062
	AS51	<0.377	1.103	0.032	0.000	0.015	<0.143	0.022	0.336	1.903	0.209
	AS52	<0.377	1.799	0.048	0.053	0.015	<0.143	0.001	<0.117	1.574	0.068
	AS53	<0.377	0.767	0.091	0.030	0.023	<0.143	0.014	<0.117	1.940	0.209
	AS54	40.426	53.957	1.473***	1.432***	5.776	8.596	0.353	41.872	49.149	14.560
	AS55	<0.377	0.911	0.024	0.000	0.019	0.151	0.000	<0.117	2.306	0.045
	AS56	<0.377	1.271	0.029	0.020	0.011	<0.143	0.000	<0.117	2.049	0.048
	AS57	7.412	4.916	0.428	0.223	0.011	18.190	0.488	2.299	2.159	0.499***
	AS58	0.582	1.775	0.132	0.225	0.015	<0.143	0.005	0.160	1.866	0.171
	AS59	17.997	93.573	1.277***	0.691***	1.729***	5.817	0.260	31.844	31.510	4.786
	AS60	<0.377	2.686	0.124	0.018	0.015	<0.143	0.000	0.336	2.306	0.099
	AS61	<0.377	1.295	0.102	0.065	0.019	<0.143	0.000	1.355***	1.610	0.055
	AS62	586.942	110.576	6.359	5.228	2.261	64.490	2.097***	75.094	117.548	12.564
	AS63	30.810	67.890	3.816	3.768	5.162	79.421	1.813***	82.359	33.413	9.599
	AS64	1.077***	1.367	0.047	0.025	0.019	<0.143	0.026	<0.117	1.903	0.082
	AS65	<0.377	1.247	0.000	0.002	0.011	<0.143	0.000	<0.117	1.720	0.041
	AS66	1.999***	4.436	0.118	0.098	0.011	1.786***	0.065	4.779	1.903	0.363
	AS67	<0.377	2.446	0.121	0.010	0.015	<0.143	0.000	0.121	1.537	0.092
	AS68	<0.377	6.402	0.039	0.015	0.011	<0.143	0.005	0.160	1.903	0.032
	AS69	0.602	7.057***	0.070	0.175	0.019	<0.143	0.000	0.252	1.757	0.018
	AS70	<0.377	6.159	0.059	0.153	0.015	<0.143	0.000	0.394	2.964	0.039
	AS71	<0.377	3.659	0.063	0.020	0.015	<0.143	0.000	0.252	2.891	0.000
	AS72	0.508	5.244	0.056	0.028	0.023	<0.143	0.000	0.174	2.049	0.011
	AS73	1.966***	2.500	0.013	0.000	0.008	<0.143	0.000	0.224	2.086	0.067
	AS74	0.495	3.232	0.125	0.135	0.012	<0.143	0.000	0.134	1.281	0.011
	AS75	<0.377	3.049	0.114	0.063	0.015	0.205	0.003	0.134	2.306	0.032
	AS76	<0.377	1.768	0.016	0.005	0.004	<0.143	0.000	0.206	1.500	0.018
	AS77	<0.377	1.707	0.032	0.013	0.000	<0.143	0.012	<0.117	1.976	0.018
	AS78	<0.377	2.500	0.023	0.055	0.008	<0.143	0.014	0.224	1.976	0.018
	AS79	<0.377	2.683	0.004	0.000	0.012	<0.143	0.016	<0.117	1.866	0.000
	AS80	0.495	2.439	0.020	0.038	0.008	0.393	0.014	<0.117	2.489	0.004
	AS81	<0.377	3.659	0.023	0.020	0.012	<0.143	0.000	0.139	1.317	0.025
	AS82	0.600***	2.683	0.018	0.015	0.004	1.864***	0.132	17.722	23.728**	2.082***
	AS83	<0.377	2.500	0.032	0.000	0.000	<0.143	0.018	0.126	1.427	0.025
	AS84	30.690	4.390	0.343	0.100	0.000	5.726	0.322	3.729	3.220	0.755**
	AS85	0.589	4.024	0.000	0.000	0.000	<0.143	0.016	<0.117	1.427	0.000
	S1	533.073	>152	6.598	7.841	12.567	157.085	4.722	337.144	114.393	17.659
	S2	633.890	150.190	6.523	8.254	10.046	>1926	6.065	1005.707	113.577	19.981
	S3	831.251	149.431	4.785	8.139	5.669	24.087	1.733***	94.102	72.421	4.110***
	S4	1338.517	148.049	8.159	8.095	2.631	128.542	4.045	351.808	116.534	9.955
	S5	755.166	146.042	6.972	8.189	12.871	110.974	3.039	1067.404	109.533	24.470
Symptomatic (n = 11)	S6	375.472	148.361	5.088	6.478	7.148	238.118	4.211	1968.340	116.126	25.185
	S7	280.991	139.086	4.962	8.356	9.513	52.265	3.114	1580.874	>118	21.434
	S8	369.730	147.291	4.554	8.361	11.901	288.574	5.294	327.996	>118	15.468
	S9	920.159	>151	6.831	8.002	7.616	13.470	0.329	79.143	113.237	4.037
	S10	492.280	104.972	4.449	4.423	8.890	1333.021	4.992	74.448	50.229	5.454
	S11	852.656	147.826	6.951	8.219	12.783	128.260	3.807	1599.047	114.902	28.328
Cutoff		1	20	1	1	1	1	1	1	20	1

Pink highlight indicates positive. ** Average of two tests, *** Average of three tests.

Supplementary FIG 1

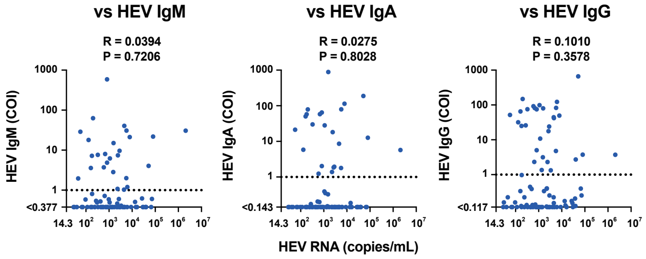

Supplementary FIG 1. Comparison between HEV RNA copies and HEV antibody titers in the asymptomatic population (n = 85). The Spearman's rank correlation coefficient was used for statical analysis.
